# Specific bFGF targeting of KIM-1 in ischemic kidneys protects against renal ischemia-reperfusion injury in rats

**DOI:** 10.1093/rb/rbac029

**Published:** 2022-05-12

**Authors:** Siqi Song, Xianglin Hou, Weiwei Zhang, Xinyu Liu, Wei Wang, Xiaoya Wang, Wenxuan Cao, Yujun Xia, Wei Chen, Chunying Shi

**Affiliations:** 1 Department of Human Anatomy, Histology and Embryology, School of Basic Medicine, Qingdao University, Qingdao 266071, China; 2 State Key Laboratory of Molecular Developmental Biology, Institute of Genetics and Developmental Biology, Chinese Academy of Sciences, Beijing 100190, China; 3 Department of Nephrology, Army Medical Center of PLA, Army Medical University, Chongqing 400038, China; 4 Department of Urology, Xinqiao Hospital, Army Medical University, Chongqing 400038, China

**Keywords:** basic fibroblast growth factor, acute kidney injury, targeted therapy, growth factor delivery system

## Abstract

Renal ischemia-reperfusion (I/R) injury is one of the major causes of acute kidney injury. However, there is still no effective treatment for this disease. Basic fibroblast growth factor (bFGF) has been reported to be beneficial for recovery from ischemic diseases. It is vital to increase the local concentration and reduce the diffusion of bFGF *in vivo* for renal I/R injury therapy. A targeted growth factor delivery system that responds to specific biological signals in the regenerative environment to guide release has been highlighted in tissue repair. In the present study, a specific peptide was fused with bFGF and called bFGF-kidney injury targeting (KIT-bFGF), and this compound specifically targeted kidney injury molecule-1 both in hypoxic renal HK-2 cells *in vitro* and ischemic kidneys *in vivo* after intravenous injection. When administered to rat models of renal I/R injury, KIT-bFGF attenuated renal tubule damage and fibrosis, and promoted functional recovery compared to the effects of native bFGF and the control. We also investigated the mechanism by which KIT-bFGF activated the ERK1/2 and Akt signaling pathways to significantly reduce apoptosis and protect against ischemic injury in the kidney. These results demonstrated that targeted delivery of KIT-bFGF could be an effective strategy for the treatment of renal I/R injury.

## Graphical Abstract

**Figure:**
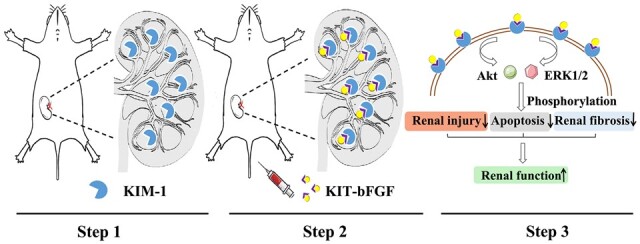

## Introduction

Acute kidney injury (AKI), which is also called acute renal failure, is characterized by rapid decreases in the metabolic function in the kidney and the glomerular filtration rate and can eventually lead to chronic kidney disease (CKD) and end-stage renal disease [1]. Renal ischemia-reperfusion (I/R) injury is the primary cause of AKI in clinical settings, and this condition destroys the structure and function of renal tubules by inducing tubular epithelial cell and vascular endothelial cell death [2, 3]. Currently, there is still no effective therapy for renal I/R.

Basic fibroblast growth factor (bFGF), also known as FGF2, is an important molecule in the FGF family. A previous study demonstrated that bFGF was effective in treating wound healing and tissue regeneration [4, 5]. After renal I/R injury, bFGF can inhibit the inflammatory response and reduce later renal fibrosis [6, 7]. bFGF can also significantly suppress apoptosis in renal tubular epithelial cells by inhibiting mitochondrial autophagy and proinflammatory signaling [8]. Currently, bFGF is administered by intravenous injection or intraperitoneal injection to treat renal I/R. However, these traditional treatments are untargeted, and the rapid diffusion of bFGF *in vivo* decreases the effective concentration in ischemic kidneys and might cause undesirable side effects.

In response to cues in the regenerative microenvironment, a targeted delivery system for growth factors is important in tissue engineering. Previously, we constructed recombinant targeted bFGF, which targeted bFGF to extracellular matrix collagen, but it was difficult to administer this recombinant bFGF by intravenous or intrarenal injection to treat ischemic kidneys. After renal injury, a series of proteins are specifically upregulated in the injured kidney. Kidney injury molecule-1 (KIM-1) is a specific protein that is markedly upregulated in proximal tubule epithelial cells immediately after toxic or renal I/R injury [9, 10]. In addition, KIM-1 is not only a sensitive signal of renal injury but also a predictive biomarker of outcomes [10]. Recently, KIM-1 has been shown to be a therapeutic target for the delivery of drugs that alleviate renal tubule injury. Tang *et al.* [11] reported a therapeutic strategy for tubule epithelial cell injury based on targeting KIM-1 by engineering red blood cell-derived extracellular vesicles to deliver small interfering RNAs in mice after I/R injury. As a result, KIM-1 serves as a useful biomarker for renal proximal tubule injury and a potential drug delivery target for treating renal injury.

Recently, the specific peptide ‘CNWMINKEC’ was shown to have a high level of binding to the KIM-1 protein *in vitro* through phage display [12]. In the present study, we fused this peptide to bFGF by gene engineering to construct bFGF that targeted the injured kidney (KIT-bFGF). Then, we explored the targeting of KIT-bFGF to hypoxic renal HK-2 cells *in vitro* and ischemic kidneys in rat renal I/R models *in vivo*. Furthermore, we investigated the protective effect of KIT-bFGF against renal I/R injury by reducing apoptosis in renal tubular cells through activation of the extracellular signal-regulated kinase (ERK1/2) and phosphatidylinositol-3-kinase (PI3K)/Akt signaling pathways.

## Materials and methods

### Purification of KIT-bFGF and native bFGF

The encoding sequence of the KIT peptide was added to bFGF complementary DNA through overlapping polymerase chain reaction. Native bFGF and KIT-bFGF were inserted into pET28a (Novagen, USA) and transformed into *Escherichiacoli* BL21. Proteins were induced in the presence of 1 mM isopropyl b-D-thiogalactopyranoside for 5 h at 37°C. After refolding, the native bFGF and KIT-bFGF recombinant proteins expressing 6× His tags were purified by nickel chromatography (GE Healthcare, UK) by AkATA primer plus system. The purity and yield of the recombinant proteins were analyzed by electrophoresis on 15% sodium dodecyl sulfate-polyacrylamide gel electrophoresis (SDS-PAGE) gel.

### KIT-bFGF and bFGF biological activity assay

The human skin fibroblasts cells (HSFs) were bought from the American Type Culture Collection (ATCC; Manassas, VA, USA) and planted at a density of 3000 cells/well in 48-well plate with Dulbecco’s modified eagle medium (DMEM; Gibco) supplemented with 10% fetal bovine serum (FBS; Gibco) and 1% penicillin–streptomycin. After 12 h, the inoculated cells were replaced with DMEM containing 2% FBS as well as stimulated with a continuous concentration of the native bFGF and KIT-bFGF for 3 days. Then, added 20 μl/well 5 mg/ml 3-(4,5-dimethylthiazol-2-yl)-2,5-diphenyltetrazolium bromide (MTT; Solarbio, M8180, China) solution to 48-well plate and incubated for 4 h at 37°C. After that, 150 μl/well of DMSO (Solarbio, China) was added and incubated for 10 min at room temperature. Finally, used microplate reader at 490 nm optical density (OD) to measure the viability of cells.

### Construction of KIT-bFGF and bFGF protein structure diagram

The sequence of model bFGF was searched in NCBI database and output with PDB files. Then, used MODELLER homology modeling methods to predict spatial structure of KIT-bFGF and native bFGF. Then, used Pymol software to show the final results.

### Conjugation of KIT-bFGF with DyLight-747

Two hundred micrograms KIT-bFGF and 200 μg native bFGF protein were, respectively, dissolved in 200 μl phosphate buffered saline packs. DyLight-747 NHS Ester was equilibrated to room temperature before using and then dissolve the DyLight-747 NHS Ester in N,N-Dimethylformamide (DMF) to a concentration of 1 mg/ml. Calculated the amount of fluorescent-labeling reagent to use for each reaction depends on the amount of protein to be labeled according to the instructions. Transferred the KIT-bFGF and native bFGF protein to be labeled and added the calculated amount of DyLight-747 NHS Ester to the reaction tube. Incubated at room temperature for 1 h, protected from light. Excess dye was filtered out using a 3k ultrafiltration tube. Discarded the excess dye and stored the labeled protein at 4°C from light for further use.

### The targeted binding of KIT-bFGF to the hypoxic HK-2 cells and survival assay

Human renal proximal tubular epithelial cell line (HK-2) was bought from the American Type Culture Collection (ATCC; Manassas, VA, USA) and cultured in DMEM (Gibco) containing 10% fetal bovine serum, and 1% penicillin–streptomycin under 5% CO_2_ and 95% humidified atmosphere at 37°C. Then, HK-2 was planted at 2.5 × 10^5^/l in 14-mm culture dish with 1% penicillin–streptomycin, serum-free and glucose-free DMEM mixed with 50 ng/ml KIT-bFGF-DyLight-747 or native bFGF-DyLight-747 and incubated in hypoxia incubator chamber for 12 h in an atmosphere of 1% O_2_, 5% CO_2_ and 94% N_2_ at 37°C, followed by reoxygenation in normal complete medium 3 h and observed under the fluorescence microscope.

HK-2 cells were planted on 48-well plate at a density of 3000 cells/well and cultured in DMEM containing 10% fetal bovine serum, and 1% penicillin–streptomycin under 5% CO_2_ and 95% humidified atmosphere at 37°C. When the cells had grown to 80%, the serum/glucose-free DMEM with a continuous concentration of the native bFGF and KIT-bFGF was replaced to culture in a hypoxia atmosphere in 37°C for 12 h. Then, reoxygenation in normal complete medium 3 h and then used MTT assay to assess HK-2 cells survival.

### Construction of renal I/R injury model in rats

All of the animal experiments were performed according to the compliance with the ‘Guide for the Care and Use of Laboratory Animals’ and all protocols were approved by the appropriate institutional committees. Male Sprague Dawley rats (200–260 g, 8–10 weeks old) were purchased from Beijing Vital River Laboratory Animal Technology Co., Ltd., and the rats were housed in a specific pathogen-free facility with the temperature of 25°C and cycle with 40–60% relative humidity in 12 h/12 h light/dark. The rats were anesthetized with 2% pentobarbital sodium (50 mg/kg) by intraperitoneal injection. After the right nephrectomy, the left renal artery was clamped for 45 min and reperfusion for 1 h. A series of experiments were performed to test the targeting ability and the protective effect of KIT-bFGF or native bFGF.

### Quantitative ELISA analysis of KIT-bFGF *in vivo*

After kidney ischemia injury and a 1 h reperfusion, KIT-bFGF (5 nmol, 0.5 mg/kg) and native bFGF (5 nmol, 0.5 mg/kg) were injected into the renal I/R rat through tail vein. At 6 and 24 h postadministration, the ischemic kidney, heart, liver, lung and serum were harvested. And then, extracted proteins and serum were analyzed using a human bFGF ELISA kit (Boster, Wuhan, China) according to the protocol.

### Fluorescent imaging of KIT-bFGF in the ischemic kidney *in vivo*

KIT-bFGF or native bFGF were fluorescently labeled by DyLight-747 NHS Ester as described above. After hypoxia injury and 1 h reperfusion, the I/R rat model was injected with 100 μl fluorescent-labeled KIT-bFGF or native bFGF proteins through the tail vein. At 6 and 24 h after administration, the distribution of fluorescein-labeled proteins was determined by *in vivo* fluorescence imaging system (Perkin Elmer, IVIS Lumina LT). Then, the animals were sacrificed and kidneys were immediately harvested to prepare frozen sections. Finally, the distribution of fluorescein-labeled proteins was observed by a fluorescence microscope.

### Assessment of renal function in rats with renal I/R injury

The animals were randomly divided into four groups: NORMAL group (sham), I/R group including KIT-bFGF group, native bFGF group and PBS group (control). Rats were anesthetized with 2% pentobarbital sodium (50 mg/kg) by intraperitoneal injection. In the I/R group, after the right nephrectomy, the left renal artery was clamped for 45 min and reperfusion for 1 h, and then KIT-bFGF (2.5 nmol, 0.25 mg/kg), native bFGF (2.5 nmol, 0.25 mg/kg) or PBS were injected through caudal vein. A series of experiments were performed to test the targeting ability and the protective effect of KIT-bFGF or native bFGF. Rats’ serum was taken at 24, 72 h, 2 and 4 weeks after administration for assessment renal function.

Renal function was assessed based on serum creatinine (Scr). Creatinine (Cr) Assay Kit (Nanjing Jiancheng, China) was used for Scr.

### Western blot analysis

The proteins of cells and kidney samples were separated, and then isolated through SDS-PAGE and transferred to PVDF membranes (Millipore, Billerica, MA, USA). The membranes were blocked with 5% skimmed milk powder for 2 h and incubated with the primary antibodies, which including KIM-1 (1:3000; Novus, Colorado, USA), cleaved Caspase-3 (1:1000; Cell Signaling, Danvers, MA, USA), Bcl-2 (1:1000; Bioworld Technology, Nanjing, China), ERK1/2 (1:2000; ZEN-BIOSCIENCE, Chengdu, China), Phospho-ERK1/2 (1:1000; ZEN-BIOSCIENCE, Chengdu, China), Akt (1:1000; ZEN-BIOSCIENCE, Chengdu, China), Phospho-Akt (1:1000; ZEN-BIOSCIENCE, Chengdu, China), FGF2 (Bioworld, Nanjing, China), Phospho-FGFR1 (1:2000; Bioss, Beijing, China), GAPDH (1:5000; Bioworld, Nanjing, China) and β-actin (1:5000; Bimake, Houston, USA) overnight at 4°C. After cleaning with TBST, the membranes were incubated with Goat Anti-rabbit HRP antibody (1:5000; Bioss, Beijing, China) at room temperature for 1 h and the signals were detected with the Omin-ECL ultra-sensitive chemiluminescence detection kit (EpiZyme, Shanghai, China). The results were analyzed by ImageJ program.

### Histochemistry assay

The isolated kidney tissues were fixed with 4% paraformaldehyde solution for 12 h. The soaked samples were embedded in paraffin and sliced into 4-µm slices. Hematoxylin-eosin staining (H&E) was performed to detect the damage of renal tubulars glomeruli. A 5-point scoring system was developed to determine tubular injury by assessing tubular epithelial necrosis, tubular dilatation, loss of brush border and cast formation, as shown below: 0-point: normal or none; 1-point: damage of tubules ≤10%; 2-point: damage of tubules 11–25%; 3-point: damage of tubules 26–45%; 4-point: damage of tubules 46–75%; 5-point: damage of tubules ≥76%. For each H&E staining sample, at last 10 contiguous areas of the cortical medulla junction and the external medulla were examined in each section. Masson’s trichrome staining was performed to detect renal fibrosis. Five blue areas of non-overlapping visual field were randomly selected from each kidney section. The results were analyzed using Image Pro-Plus v. 6.0 software (Bethesda, MD, USA).

TUNEL (TdT Mediated dUTP Nick End Labeling) apoptosis Detection Kit (Alexa Fluor 488) (Yeasen, Shanghai, China) was used to detect nuclear DNA breaks during late apoptosis. In apoptotic nucleus, TUNEL-positive nucleus (green) was labeled by fluorescein 488. Each slice randomly selected five fields at ×200 original magnification for quantitative of TUNEL-positive cells. The number of fluorescein-labeled positive cells divided by the total number of cells (DAPI) and multiplied by 100 was the positive rate of apoptotic cells. And anti-cleaved Caspase-3 antibody (1:700; Cell Signaling, Danvers, MA, USA) was used to detect apoptosis, Paraffin-embedded sections of kidney tissue were dewaxed with xylene and then dehydrated with gradient concentration alcohol. After blocking with serum, the kidney sections were incubated with cleaved Caspase-3 antibody (1:700; Cell Signaling, Danvers, MA, USA) overnight at 4°C, followed by the incubation with secondary antibody and then stained with 3,3′-diamino-benzidine and hematoxylin. Finally, neutral resin adhesive was used to permanently fix the sections. The cleaved Caspase-3 expression was assessed by the product of the positive cell expression area and stain intensity. The positive cells expression area was scored: 0-point (positive cells expression: 0%); 1 point (positive cells expression: 1–25%); 2-point (positive cells expression: 26–50%); 3-point (positive cells expression: 51–75%); 4-point (positive cells expression: 76–100%); The staining intensity of positive cells was scored: 0-point: colorless; 1-point: light yellow; 2-point: Brown; 3-point: Tan. Ten fields were randomly selected for each slice, and all results were repeated three times.

### Immunofluorescence staining

Immunofluorescence staining was performed on paraffin sections as the same as H&E staining slices. The primary antibodies included rabbit anti-KIM-1 (1:200; Novus, CO, USA) and mouse anti-FGF2 (1:200; Santa Cruz Biotechnology, USA). Both primary antibodies were mixed and incubated together overnight at 4°C. The slices were exposed to Goat polyclonal Secondary Antibody to Rabbit (Alexa Fluor^®^ 594, 1:500; Abcam, USA) and Goat polyclonal Secondary Antibody to Mouse (Alexa Fluor^®^ 488, 1:500; Abcam, USA) at room temperature for 1 h. Then the sections were observed using fluorescence microscopes.

### Statistical analysis

All the data are expressed as the mean ± standard deviation (SD). GraphPad Prism v. 8.00 was used to analyze the statistics and comparisons between multiple groups were performed using one-way ANOVA. Student's *t*-test was performed with SPSS. One-way ANOVA was used for renal function data, H&E data, Masson data, TUNEL data and cleaved Caspase-3 data. Student's *t*-test was used for the bioactivity of KIT-bFGF and native bFGF, and HK-2 cell survival. The level of *P *<* *0.05 represented statistical significance. Data were expressed as mean ± SD.

## Results

### KIT-bFGF specifically bound to hypoxic HK-2 cells *in vitro*

**Figure 1.**
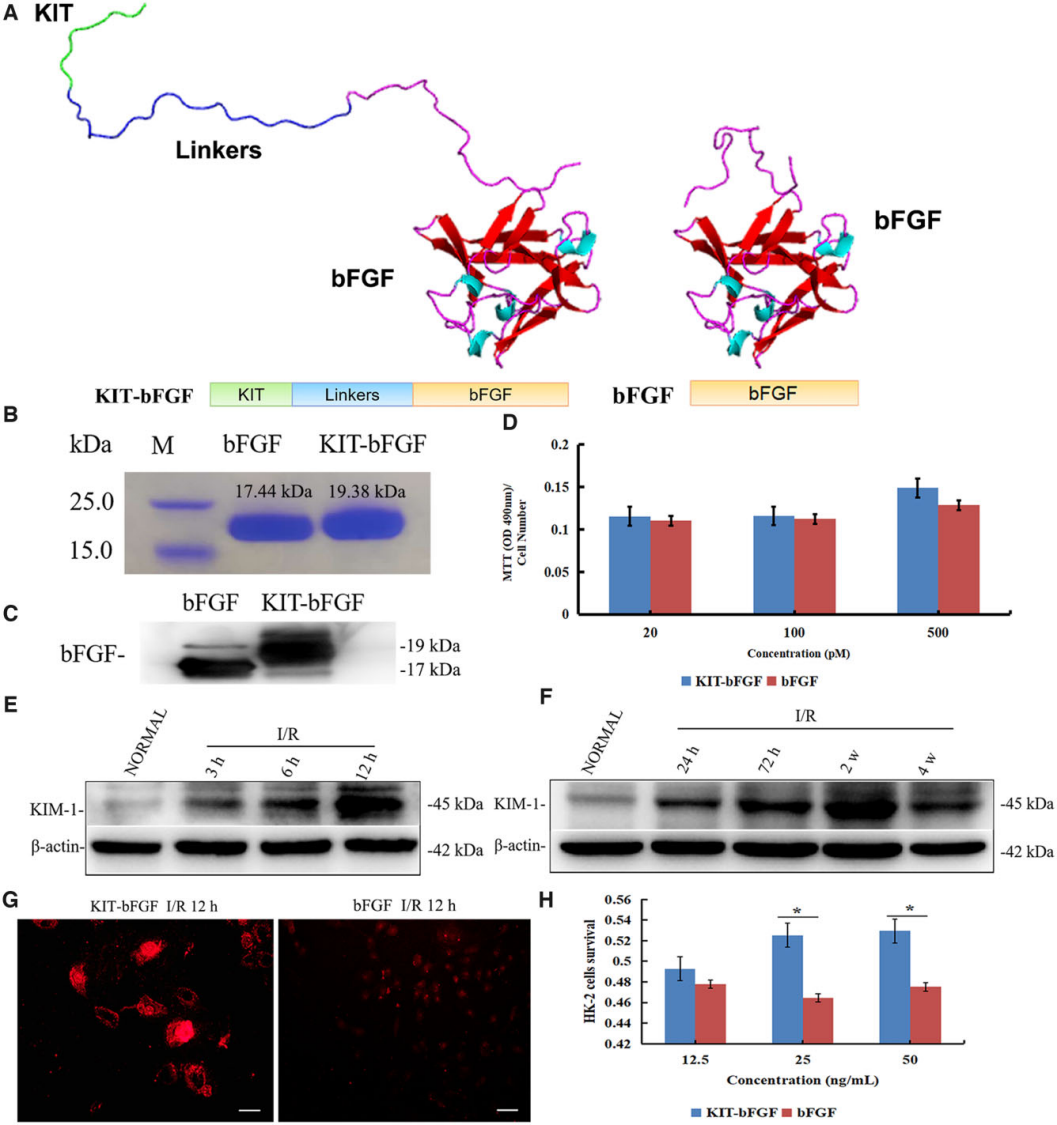
Purification, bioactivity and targeting capacity of KIT-bFGF. (**A**) Protein structure prediction of recombinant KIT-bFGF and native bFGF. (**B**) Purified KIT-bFGF and native bFGF were detected on the SDS-PAGE gel. (**C**) Purified KIT-bFGF and native bFGF were detected by western blot. (**D**) The bioactivity of KIT-bFGF and native bFGF was detected by MTT assay through the promotion of HSFs proliferation. (**E**) The expression of KIM-1 in HK-2 cells after I/R injury *in vitro* by western blot. (**F**) The expression of KIM-1 in ischemic kidneys after I/R injury *in vivo* by western blot. (**G**) Fluorescent observation of targeting capacity of the KIT-bFGF and bFGF proteins labeled with DyLight-747 dye after HK-2 I/R injury for 12 h. The scale bar = 50 μm. (**H**) Cell survival was detected by MTT after KIT-bFGF and bFGF treatment in HK-2 cells after I/R injury *in vitro.* *P <* *0.05.

The specific peptide (CNWMINKEC), also known as the KIT peptide, was fused with the carboxy terminus of native bFGF to construct modified KIT-bFGF. Then, protein structure prediction was performed by MODELLER homology modeling, and the results showed that the fusion of the KIT peptide did not affect the active domains of bFGF (Fig. 1A). Native bFGF and KIT-bFGF were purified, and the expression of both proteins was examined by SDS-PAGE (Fig. 1B) and western blotting (Fig. 1C). Then, we compared the biological activity of bFGF and KIT-bFGF, as shown in Fig. 1D. Both of the bFGFs had similar bioactivities and promoted the proliferation of HSFs, which indicated that the fusion of the KIT peptide did not affect the bioactivity of bFGF.

It was reported that KIM-1 is upregulated dramatically after renal injury [9]. Therefore, we examined the expression of KIM-1 after I/R injury in both HK-2 cells and the kidney. The results showed that KIM-1 was significantly increased after I/R injury both *in vitro* and *in vivo* (Fig. 1E and F). Then, bFGF and KIT-bFGF were labeled with DyLight-747 to determine the binding capacity of KIT-bFGF for HK-2 cells under hypoxic conditions. As shown in Fig. 1G, the fluorescence intensity in the KIT-bFGF group was much stronger than that in the bFGF group. This result demonstrated that more KIT-bFGF could bind to hypoxic HK-2 cells. Moreover, the cell morphology in the KIT-bFGF group was more intact than that in the bFGF group, indicating the potential protective effect of KIT-bFGF against hypoxic damage. In addition, the effect of KIT-bFGF on the survival of HK-2 cells under I/R conditions *in vitro* was examined by MTT assay. As shown in Fig. 1H, the survival of HK-2 was similar in both the KIT-bFGF and bFGF groups at low concentrations (12.5 ng/ml). As the concentration increased (25, 50 ng/ml), cell survival significantly increased in the KIT-bFGF group compared to the bFGF group. Thus, these results showed that KIT-bFGF could specifically target hypoxic HK-2 cells and protect these cells from I/R injury *in vitro*.

### KIT-bFGF targeted and was retained in the ischemic kidney *in vivo* after intravenous injection

**Figure 2.**
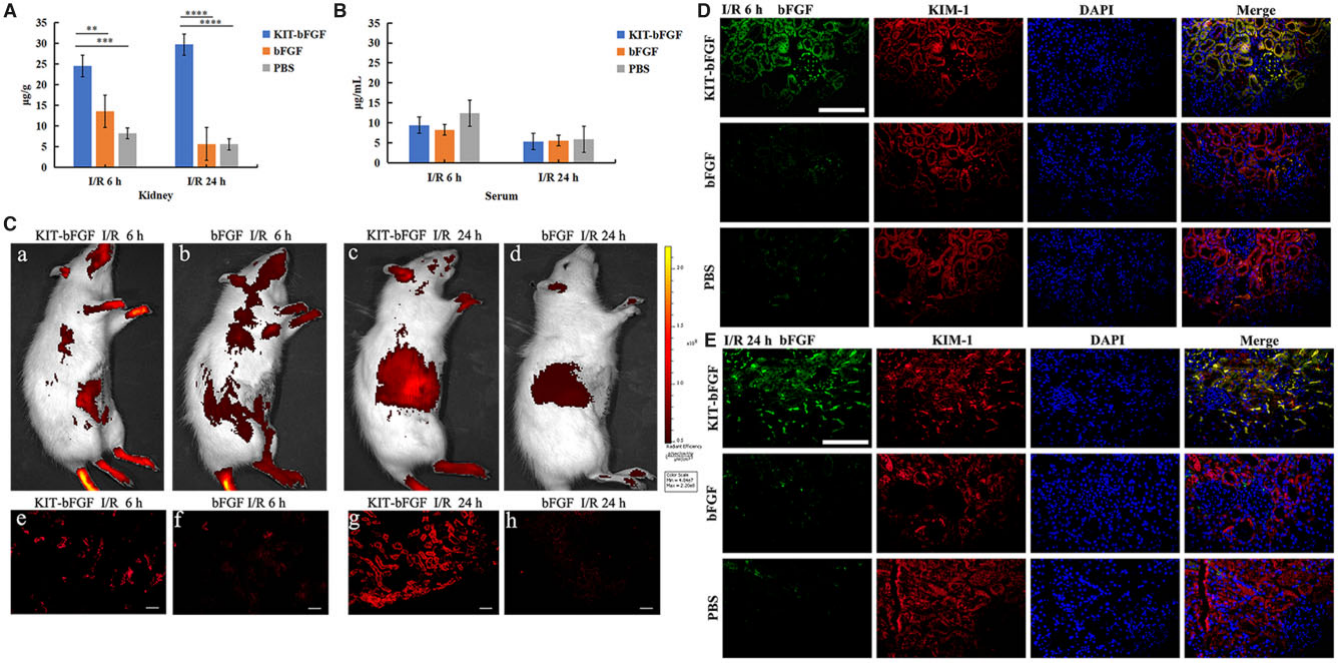
KIT-bFGF could target to the ischemic kidney and retain in the kidney after renal I/R injury. (**A**) Quantitative ELISA assay for bFGF in kidney at 6 and 24 h postinjection. At 6 h postinjection, KIT-bFGF = 24.507 ± 2.002 μg/g, bFGF = 13.523 ± 3.704 μg/g and PBS = 8.208 ± 1.562 μg/g. At 24 h postinjection, KIT-bFGF = 29.696 ± 0.682 μg/g, bFGF = 5.618 ± 0.266 μg/g and PBS = 5.572 ± 0.746 μg/g. Data are presented as mean ± SD. *N* = 6, **P <* *0.01, ***P <* *0.001, ****P <* *0.0001. (**B**) Quantitative ELISA assay for bFGF in serum. At 6 h postinjection, KIT-bFGF = 9.407 ± 1.849 μg/ml, bFGF = 8.214 ± 0.974 μg/ml and PBS = 12.399 ± 4.141 μg/ml. At 24 h postinjection, KIT-bFGF = 5.315 ± 0.292 μg/ml, bFGF = 5.554 ± 0.410 μg/ml and PBS = 5.873 ± 0.464 μg/ml. Data are presented as mean ± SD. *N* = 6. (**C**, a–d) Animals' imaging for fluorescence DyLight-747 dye labeled KIT-bFGF and bFGF at 6 and 24 h postinjection *in vivo* by fluorescence imaging system. Ex/Em = 748/771. (e–h) Fluorescence distribution in kidneys after 6 and 24 h administration. Scale bar = 50 μm. *N* = 6. (**D, E**) Immunofluorescence staining for bFGF (green) and KIM-1 (red) colocalization (yellow) at 6 and 24 h postadministration. Scale bar = 20 μm. Each slice representative of ×400 original magnification. *N* = 6.

After we confirmed the binding of KIT-bFGF to hypoxic HK-2 cells *in vitro*, we further explored the capacity of KIT-bFGF to target ischemic kidneys *in vivo*. Renal I/R injury models were constructed, and then KIT-bFGF or bFGF was administered by intravenous injection. ELISA was used to examine the retention of KIT-bFGF in the ischemic kidney or serum at 6 and 24 h after intravenous injection. As shown in Fig. 2A, the KIT-bFGF content in the ischemic kidney was significantly higher than that in the native bFGF and PBS groups at 6 and 24 h postinjection. However, the amount of KIT-bFGF in the serum was not significantly different from that in the native bFGF and PBS groups at 24 h after injection (Fig. 2B). These results suggested that increased KIT-bFGF was retained in ischemic kidneys and had reduced diffusion in peripheral blood. Additionally, we examined the metabolism of KIT-bFGF and native bFGF in heart, liver and lung tissues. As shown in Supplementary Fig. S1B and C, the amount of KIT-bFGF and native bFGF in liver were slightly higher compared to PBS group, but much lower than those in ischemic kidneys, especially at 24 h postadministration. The amount of KIT-bFGF in heart and lung tissues was much lower than PBS group at 6 h postadministration, and then was similar with PBS group after 24 h administration.

To further explore the capacity of KIT-bFGF to target ischemic kidneys *in vivo*, DyLight-747-labeled bFGF and KIT-bFGF were used to examine homing potential (Supplementary Fig. S1A). At 6 and 24 h after injection, significant fluorescent signals could be observed in the region of the kidney in the KIT-bFGF group by an *in vivo* fluorescence imaging system (Fig. 2C, a–d). These results were further confirmed by gross anatomical examination, and the kidneys were prepared as frozen sections. As shown in Fig. 2C (e–h), the fluorescence intensity in the KIT-bFGF group was much stronger than that in the bFGF group at 6 and 24 h after injection. In addition, significant fluorescent signals were retained in renal tubules where KIM-1 was upregulated in the KIT-bFGF group, whereas the fluorescent signals were weak and the location was indistinct in the bFGF group. These results suggested that KIT-bFGF could specifically target and be retained in ischemic kidneys in renal I/R injury *in vivo* after intravenous injection. To determine whether KIT-bFGF could specifically target KIM-1 in ischemic kidneys, immunofluorescence staining was used to determine the colocalization of KIT-bFGF and KIM-1 in ischemic kidneys. As shown in Fig. 2D and E and Supplementary Fig. S2, the fluorescence intensity of bFGF in the KIT-bFGF group was significantly higher than that in the native bFGF and PBS groups (green), and the expression of KIM-1 was not significantly different among the three groups (red). Most bFGF was colocalized with KIM-1 in the KIT-bFGF group (yellow) at 6 and 24 h postadministration compared to the bFGF and PBS groups, and there was a significant difference. These results suggested that KIT-bFGF could effectively target KIM-1 and be retained in the ischemic kidney after I/R injury *in vivo.*

### KIT-bFGF improved the recovery of renal function after acute renal I/R injury

**Figure 3.**
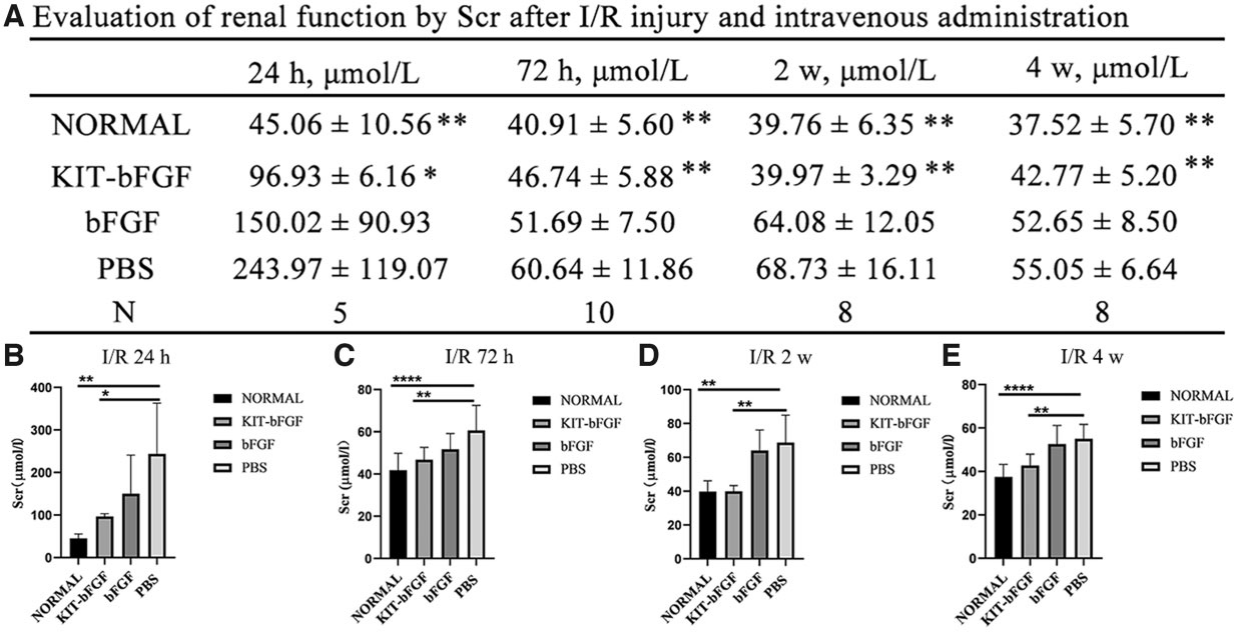
KIT-bFGF improved the recovery of renal function after acute renal I/R injury. (**A**) Summary of Scr data for renal function after administration. *P <* *0.05. **P <* *0.01. Data are presented as mean ± SD (**B–E**) Histogram of Scr assay for renal function after I/R injury and at 24, 72 h, 2 and 4-week postadministration. *P <* *0.05, **P <* *0.01, ****P <* *0.0001.

We determined whether KIT-bFGF effectively protects ischemic kidneys after I/R injury. After constructing I/R injury models, renal function and pathological outcomes were evaluated at 24, 72 h, 2 and 4 weeks after the administration of KIT-bFGF, native bFGF and PBS. Scr levels were examined to determine the glomerular filtration function of the kidney (Fig. 3). The level of Scr in the KIT-bFGF group (96.93 ± 6.16) was significantly lower than that in the bFGF (150.02 ± 90.93) and PBS (243.97 ± 119.07) groups at 24 h after intravenous injection (*P *<* *0.05). Over time, there was a certain degree of recovery in all groups, but the level of Scr in the KIT-bFGF group was still significantly different from that in the two other groups (*P *<* *0.01). These results revealed that due to targeted delivery, KIT-bFGF could effectively improve the recovery of renal function compared to that in the bFGF and PBS groups after acute I/R injury.

### KIT-bFGF protected the kidney against acute renal I/R injury

**Figure 4.**
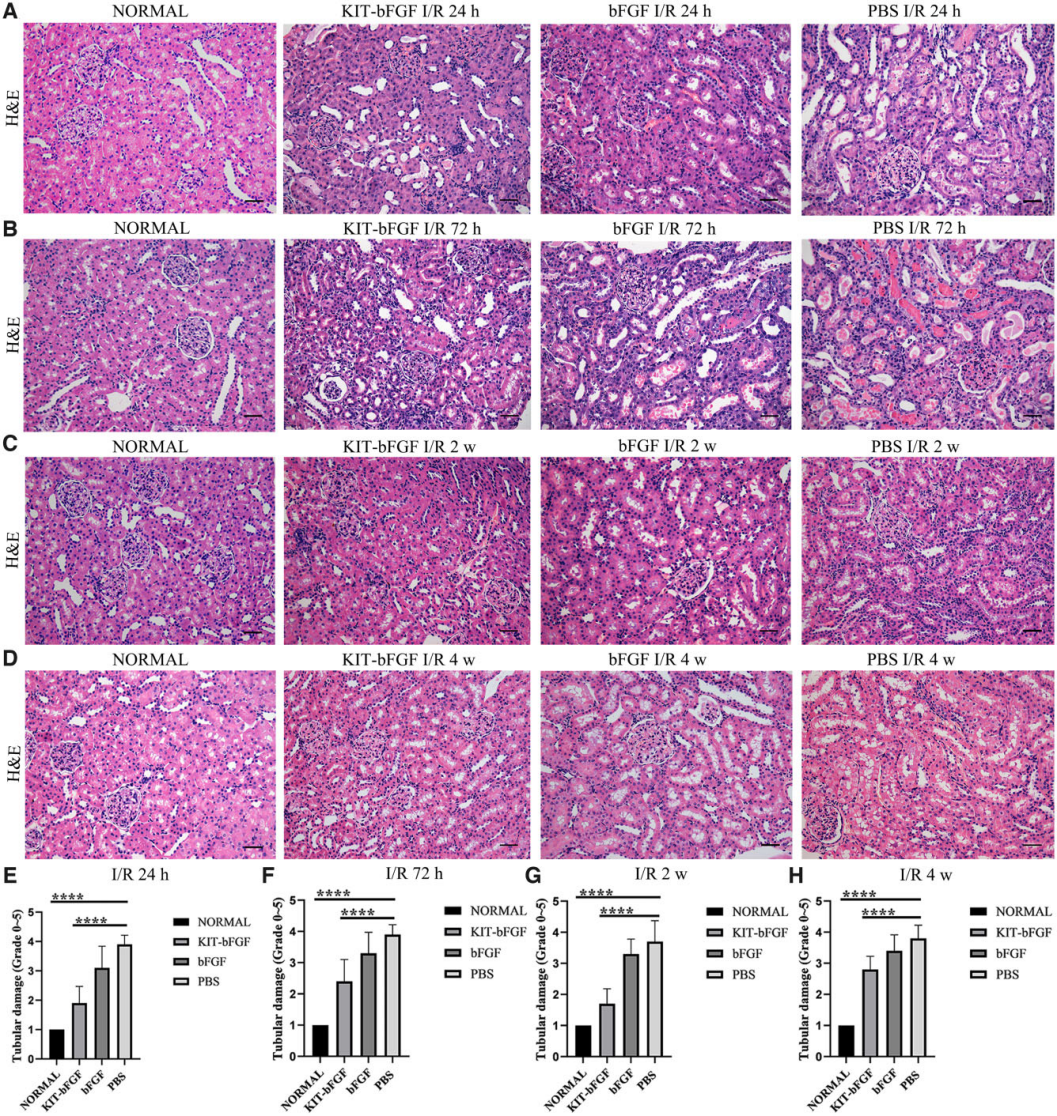
KIT-bFGF protected kidney against acute renal I/R injury. (**A–D**) Histopathological staining (H&E) for kidney pathological injury observation. Each slice representative of ×200 original magnification. Scale bar = 50 μm. (**E–H**) Quantitative analysis of renal tubule injury based on H&E staining. All data are expressed as mean ± SD. ****P <* *0.0001. *N* = 8.

Then, pathological examinations were evaluated at 24, 72 h, 2 and 4 weeks after injection. Histopathological staining (H&E staining) was used to assess renal morphological damage. As shown in Fig. 4, there was no apparent renal damage in the normal group, whereas control PBS-injected rats showed severe renal damage, cast formation, abundant tubular epithelial necrosis and increased tubular dilatation and congestion. In addition, the tubules exhibited massive proteinuria, and the glomerulus was heavily congested, whereas treatment with KIT-bFGF and bFGF decreased tubule damage, congestion and cast formation. Rats treated with KIT-bFGF had the least damage to renal tubules; most tubules had intact shapes and reduced congestion among the three treatment groups. The renal tubular injury scores also showed that treatment with KIT-bFGF (1.90 ± 0.57, 2.40 ± 0.70, 1.70 ± 0.48, 2.80 ± 0.42) markedly reduced the renal damage scores compared to bFGF (3.10 ± 0.74, 3.30 ± 0.68, 3.30 ± 0.48, 3.40 ± 0.52) and PBS (3.90 ± 0.32, 3.90 ± 0.32, 3.70 ± 0.68, 3.80 ± 0.42) at 24, 72 h, 2 and 4 weeks after intravenous injection. These results demonstrated that KIT-bFGF could effectively protect the kidney against renal I/R injury.

### KIT-bFGF attenuated the renal fibrosis after acute renal I/R injury

**Figure 5.**
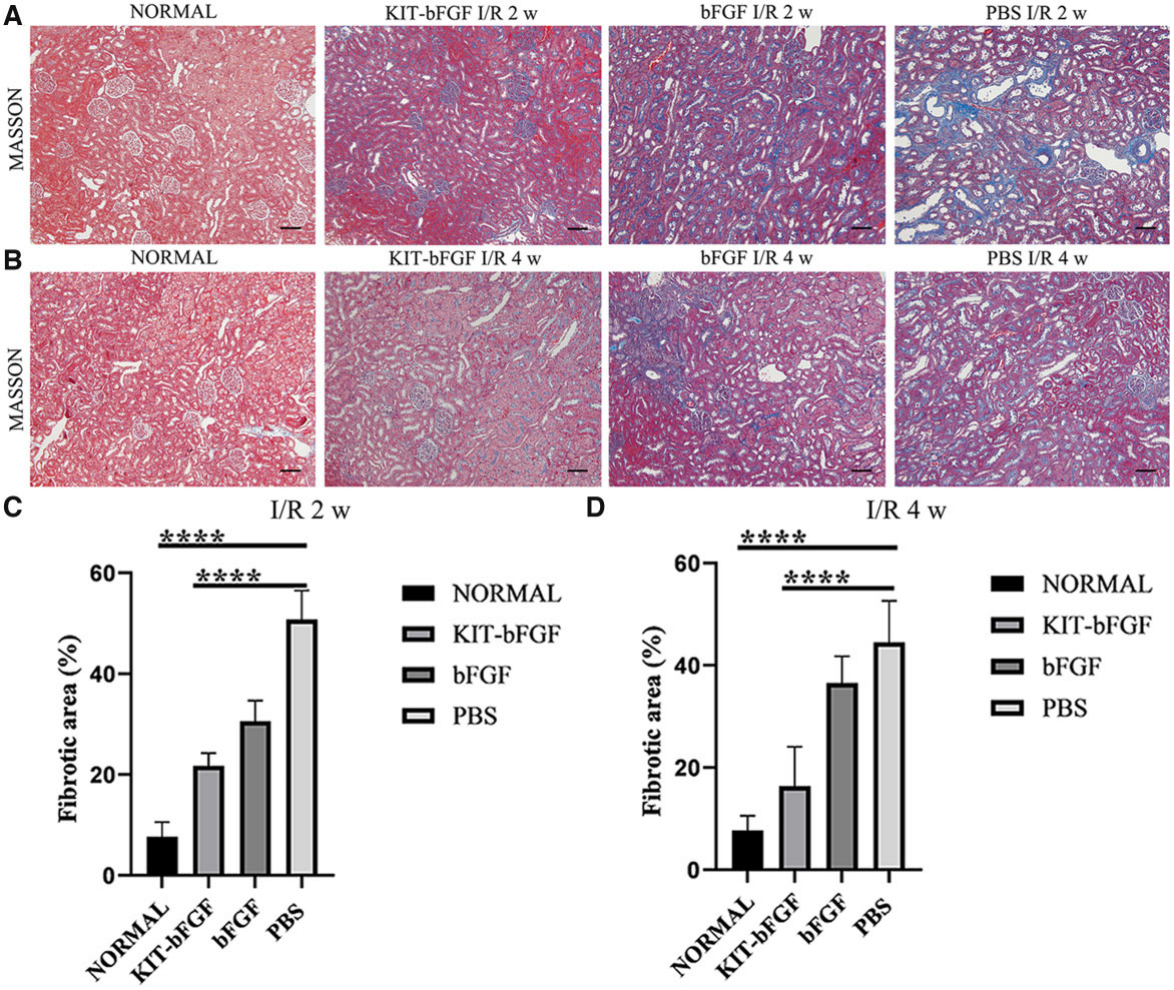
KIT-bFGF attenuated the renal fibrosis after acute renal I/R injury. (**A, B**) Blocked renal interstitial volume (blue staining and octothorpe) assay by using Masson’s trichrome staining. ×100 original magnification. Scale bar = 100 μm. (**C, D**) Quantitative analysis of the area of renal interstitial fibrosis in obstructed kidneys. All data are expressed as mean ± SD. ****P <* *0.0001. *N* = 8.

It was reported that the progressive loss of kidney function until the terminal stage was a typical characteristic of CKD. The most significant manifestation was morphological changes, including tubular fibrosis and glomerular sclerosis. Both conditions lead to progressive destruction and irreversible damage to the kidneys [6, 13]. Therefore, Masson’s trichrome staining was used to assess renal fibrosis at 2 and 4 weeks after injection (Fig. 5). No apparent renal fibrosis was observed in the normal (7.74 ± 2.83%) group. Treatment with KIT-bFGF (21.80 ± 2.47%, 16.36 ± 7.72%) significantly attenuated renal fibrosis (blue staining), and the fibrotic area was significantly smaller than that in the bFGF-treated (30.62 ± 4.03%, 36.59 ± 5.20%) and PBS-treated (50.76 ± 5.77%, 44.51 ± 8.11%) groups. Thus, KIT-bFGF played an important role in decreasing renal fibrosis after renal I/R injury.

### KIT-bFGF protected against renal I/R injury by inhibiting renal tubular epithelial cell apoptosis through regulating proapoptotic proteins

**Figure 6.**
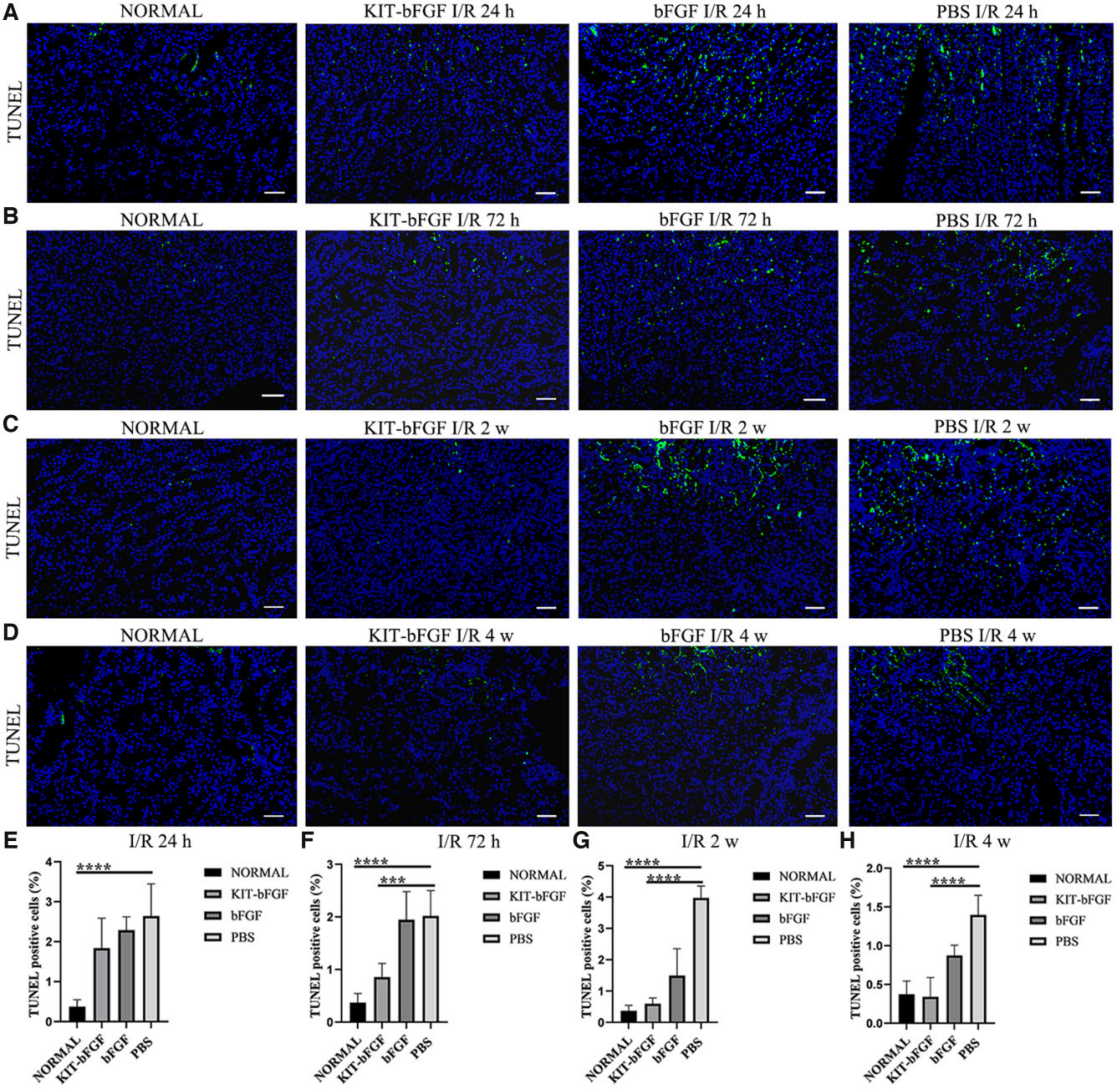
KIT-bFGF inhabited the apoptosis through the regulation of proapoptotic proteins. (**A–D**) TUNEL staining for apoptosis cells assessment (green). ×200 original magnification, scale bar = 50 μm. (**E–H**) Quantitative of kidney TUNEL-positive cells. All data are expressed as mean ± SD. ***P <* *0.001, ****P <* *0.0001. *N* = 8.

TUNEL staining was used to determine the number of apoptotic cells after renal I/R injury (Fig. 6). The results showed that TUNEL-positive cells in the KIT-bFGF group (1.84 ± 0.75%, 0.86 ± 0.26%, 0.60 ± 0.18%, 0.34 ± 0.25%) significantly decreased compared to those in the bFGF group (2.29 ± 0.33%, 1.95 ± 0.54%, 1.50 ± 0.86%, 0.87 ± 0.13%) and PBS group (2.64 ± 0.80%, 2.02 ± 0.48%, 3.97 ± 0.38%, 1.40 ± 0.25%) at 24, 72 h, 2 and 4 weeks after intravenous injection. This finding revealed the protective effect of KIT-bFGF against renal I/R injury.

**Figure 7.**
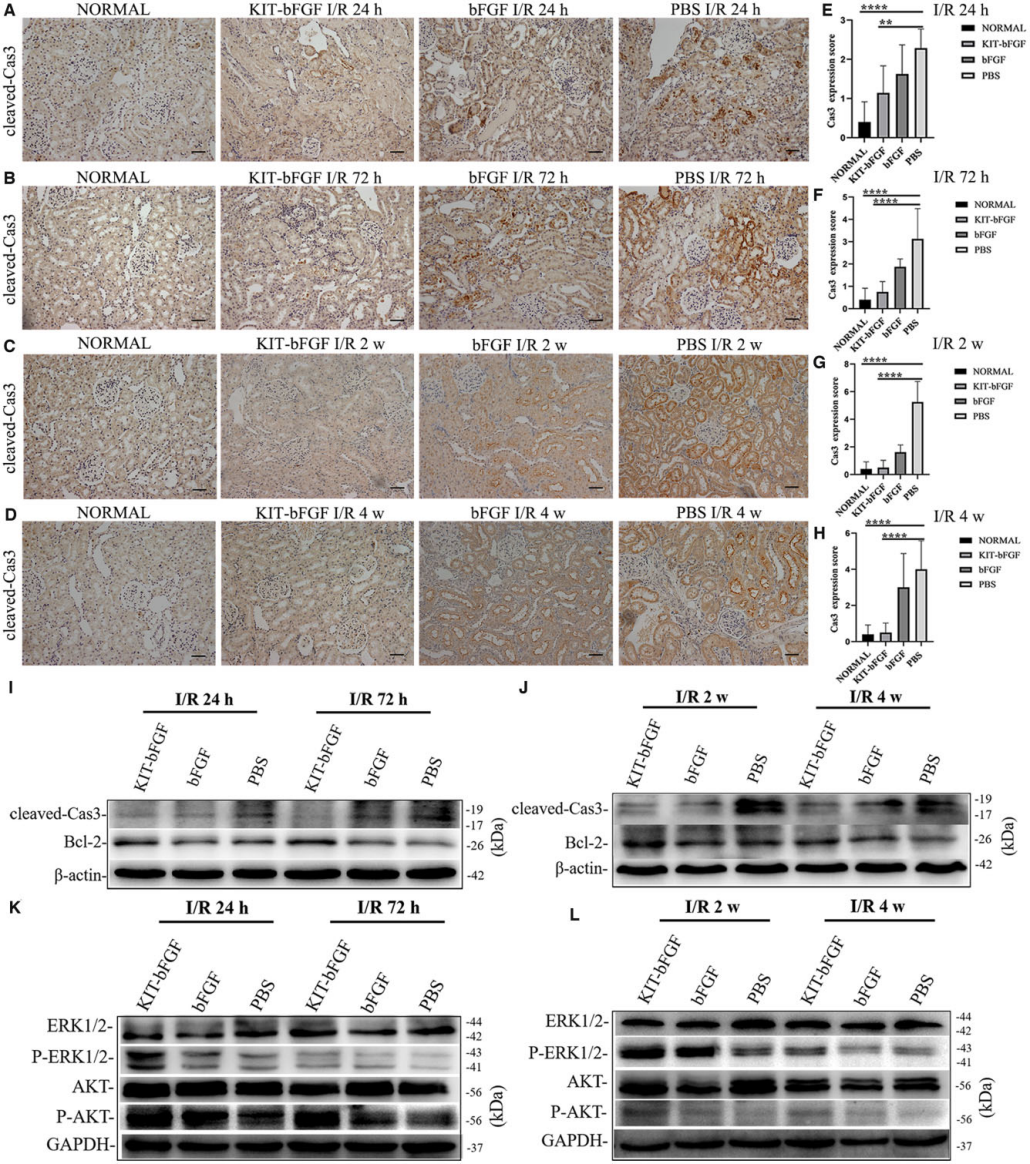
KIT-bFGF inhabited the apoptosis through the regulation of proapoptotic proteins. (**A–D**) IHC staining for cleaved-Caspase3 after I/R injury and administration. ×200 original magnification, scale bar = 50 μm. (**E–H**) Quantitative of IHC staining for cleaved caspase-3. All data are expressed as mean ± SD. **P <* *0.01, ****P <* *0.0001. *N* = 8. (E) At 24-h postadministration: NORMAL = 0.40 ± 0.52, KIT-bFGF = 1.14 ± 0.69, bFGF = 1.63 ± 0.74, PBS= 2.29 ± 0.49. (F) At 72-h postadministration: KIT-bFGF = 0.75 ± 0.46, bFGF = 1.88 ± 0.35, PBS= 3.13 ± 1.36. (G) At 2-week postadministration: KIT-bFGF = 0.50 ± 0.53, bFGF = 1.63 ± 0.52, PBS= 5.25 ± 1.49. (H) At 4-week postadministration: KIT-bFGF = 0.50 ± 0.53, bFGF = 3.00 ± 1.87, PBS= 4.00 ± 1.58. (**I–L**) Western blot assayed the expression of cleaved Caspase-3, Bcl-2, ERK1/2, phospho-ERK1/2, Akt and phospho-Akt at 24, 72 h, 2-and 4-week postadministration. β-actin and GAPDH were used as a control.

We further demonstrated that the TUNEL-positive signal was due to apoptosis after renal I/R injury. Caspase-3 is the most important terminal cleavage enzyme associated with apoptosis and is activated as cleaved Caspase-3 during the early stage of apoptosis [14, 15]. We examined cleaved Caspase-3 expression by immunohistochemical staining at 24, 72 h, 2- and 4-week postadministration. We observed that both normal and KIT-bFGF-treated rats showed reduced expression of cleaved Caspase-3, whereas bFGF-treated and PBS-treated rats showed increased expression of cleaved Caspase-3 (Fig. 7). The level of cleaved Caspase-3 expression was quantified and showed that KIT-bFGF strikingly decreased cleaved Caspase-3 expression, and these levels gradually approached those of the normal group at 2 and 4-week postadministration. In contrast, cells treated with bFGF and PBS showed much higher cleaved Caspase-3 expression (Fig. 7C, D, G and H). Then, western blot analysis was used to evaluate the expression of cleaved Caspase-3 and Bcl-2 to verify whether KIT-bFGF was involved in apoptosis at 24, 72 h, 2 and 4 weeks after administration. The level of cleaved Caspase-3 was significantly increased, whereas the level of Bcl-2 was reduced in bFGF-treated and PBS-treated rats. The expression of cleaved Caspase-3 was suppressed by treatment with KIT-bFGF (Fig. 7I–J). Therefore, these results showed that KIT-bFGF inhibited apoptosis by regulating proapoptotic proteins.

It was reported that bFGF protects against renal I/R injury by activating the ERK1/2 and Akt pathways to alleviate renal tubular apoptosis [7]. To further determine the molecular mechanism of KIT-bFGF in kidney protection, we examined the phosphorylation of ERK1/2 and Akt in the ischemic kidneys at 24, 72 h, 2- and 4-week postadministration. As shown in Fig. 7K and L, treatment with both KIT-bFGF and native bFGF induced the expression of phospho-ERK1/2 and phospho-Akt. Treatment with KIT-bFGF showed prolonged activation of ERK1/2 and Akt compared to native bFGF at 24, 72, 2 and 4 weeks after I/R injury. Furthermore, the expression levels of phospho-ERK1/2 and phospho-Akt were significantly reduced in the PBS group compared to those in the KIT-bFGF group, which indicated that the ERK1/2 and Akt pathways might be involved in the protective effects of KIT-bFGF.

## Discussion

Stimuli-responsive delivery systems have attracted growing interest in the development of drug delivery systems, tissue engineering and biomedical devices, and these systems are sensitive to regenerative signals or pathological abnormalities in the microenvironment. In tissue engineering, these targeted delivery systems mainly focus on the spatial and/or temporal release of growth factors in specific regions to promote tissue regeneration [16]. A series of stimuli-responsive delivery systems were constructed and were capable of responding to specific biological signals in a regenerative environment to trigger release [17]. Except for biomaterial scaffolds, the modification of growth factors was an alternative strategy that was efficient and facilitated low-dose repeated administration [18]. In a previous study, we constructed an ischemic myocardium targeting system known as IMT-VEGF to guide vascular endothelial growth factor (VEGF) to the ischemic myocardium. IMT-VEGF could interact with cardiac troponin I, which is specifically upregulated in the ischemic myocardium. When IMT-VEGF was intravenously injected into rats and pigs with MI, it was significantly concentrated in the ischemic heart and promoted angiogenesis and cardiac function [19]. Lung endothelial cell-targeted bFGF was reported to guide bFGF to pulmonary vascular endothelial cells and promote regeneration after radiation-induced lung injury [20]. In this study, a novel modified KIT-bFGF was constructed and specifically targeted the ischemic kidney, which effectively attenuated renal I/R injury and fibrosis in rats.

A series of studies have reported that bFGF can effectively protect against renal injury and fibrosis by attenuating apoptosis [21] and accelerating tissue repair [13]. To increase the regional concentration of bFGF and decrease its diffusion, a specific targeting version of bFGF was constructed that interacted with KIM-1 and guided bFGF to bind to ischemic renal tissues after intravenous injection. The peptide ‘CNWMINKEC’ was fused with bFGF by gene engineering, and we examined the targeting capacity of KIT-bFGF *in vitro* and *in vivo*. As shown in Figs 1G and 2C, DyLight-747 labeled KIT-bFGF and bFGF showed significant difference fluorescent signals on the surface of hypoxic HK-2 cells and in ischemic renal tissues. Furthermore, the level of bFGF in kidney, serum and other tissues was assessed by ELISA, and the results showed that more bFGF was retained in ischemic kidneys than in other tissues, as well as bFGF was less diffuse in the blood in the KIT-bFGF group than in the other groups (Fig. 2A and B and Supplementary Fig. S1B and C). These results indicated that KIT-bFGF could specifically target and be retained in hypoxic renal tubule cells and ischemic renal tissues.

Then, the renal protective effects of this modified protein were assessed. As shown in Fig. 3, although the levels of Scr decreased in both the KIT-bFGF and native bFGF groups compared to the PBS group, the KIT-bFGF group showed a marked reduction compared with the PBS group at different times after injection. Pathological staining showed that the lumen of renal tubules exhibited significantly reduced apoptosis, morphological damage was alleviated and the renal fibrosis area was markedly reduced in the KIT-bFGF group. These results were consistent with renal function analysis, which demonstrated that the targeted delivery of bFGF to ischemic kidneys by KIT-bFGF could attenuate I/R injury and effectively promote the recovery of renal function (Figs 4–7).

Many studies have reported that AKI remains a common disease with a high mortality rate and is mainly caused by I/R injury. The pathological mechanism of AKI may include inflammation, autophagy, apoptosis, oxidative stress, mitochondrial damage and endoplasmic reticulum (ER) stress [22]. However, there is still no definitive treatment for renal I/R injury, and regenerative medicine provides prospective therapeutic strategies. Unlike cerebral and myocardial ischemia, which are difficult to recover from, the kidney has complex architecture and the ability to recover both morphologically and functionally. In renal epithelial cells, especially those in the S3 segment of the proximal tubule, are susceptible to ischemic and toxic injury [23]. Protecting these cells is crucial for AKI therapy. And the mechanism by which KIT-bFGF protects against renal I/R injury was also explored. Tan *et al.* revealed that bFGF could reduce mitochondrial-damaging parameters and the HMGB1-mediated inflammatory response caused by renal I/R injury [24]. In addition, bFGF also inhibited renal tubular epithelial cell apoptosis and suppressed extensive ER stress by activating the PI3K/Akt and MEK-ERK1/2 signaling pathways after I/R injury [7]. Hence, we examined the expression of Bcl-2, cleaved Caspase-3, phospho-ERK1/2 and phospho-Akt to determine the role of KIT-bFGF in renal injury recovery. The results showed that the expression of cleaved Caspase-3 markedly decreased compared to that in the native bFGF and PBS groups, whereas the expression of Bcl-2, phospho-ERK1/2 and phospho-Akt showed increased after 24, 72 h, 2- and 4-week administration (Fig. 7I–L). Based on these results, we proposed that KIT-bFGF attenuated renal tubular epithelial cell apoptosis through prolonged activation of the ERK1/2 and Akt pathways.

In addition, the transmembrane glycoprotein KIM-1 is a target protein of KIT-bFGF. In the normal mammalian kidney, KIM-1 expression is rare but is abundantly induced in injured proximal tubular epithelial cells after acute renal injury [25]. As a result, KIM-1 is considered as a sensitive and specific marker of renal tubular epithelial injury and serves as an ideal target for the targeted delivery of drugs in AKI therapy. Recently, the function of KIM-1 was investigated further. KIM-1 is a receptor that mediates phagocytosis of apoptotic cells and oxidized lipids. However, sustained expression of KIM-1 directly promotes progressive interstitial kidney inflammation and kidney fibrosis. This process was associated with the upregulation of the proinflammatory cytokine monocyte chemotactic protein-1 and activation of rapamycin (mTOR) or the MAPK pathway [25–28]. Additionally, the low-avidity anti-Tim-1 antibody RMT1-10 was injected to antagonize KIM-1, and RMT1-10 accumulated in proximal tubules where KIM-1 is expressed. The binding of RMT1-10 to KIM-1 could mediate the activation of immune cells in the glomerulonephritis kidney model [29].

In summary, a specific bFGF delivery system was constructed to control the release of bFGF in response to KIM-1 in the ischemic kidney. This delivery system could specifically identify and be retained by hypoxic renal HK-2 cells *in vitro* and ischemic kidneys *in vivo* after intravenous injection. Treatment with KIT-bFGF in rat models with renal I/R injury could attenuate renal tubule damage, renal fibrosis, cell apoptosis and effectively improved functional recovery at different stages. These results are encouraging and provide a potential therapeutic strategy for the delivery of bFGF in renal I/R injury.

## Supplementary data

Supplementary data are available at *REGBIO* online.

## Funding

This study is supported by the National Natural Science Foundation of China (81970590 and 31670989) and the Key Research and Development Program of Shandong Province (2019GSF107037).

*Conflicts of interest statement*. The authors declare that the research was conducted in the absence of any commercial and the authors have no competing financial interests.

## Supplementary Material

rbac029_Supplementary_Data
