# Supplementary material for: Specific bFGF targeting of KIM-1 in ischemic kidneys protects against renal ischemia-reperfusion injury in rats
Source: Regen Biomater. 2022 May 12;9:rbac029. doi: 10.1093/rb/rbac029 (PMC9127338; doi:10.1093/rb/rbac029)
Supplement: rbac029_Supplementary_Data [file rbac029_supplementary_data.docx]

**
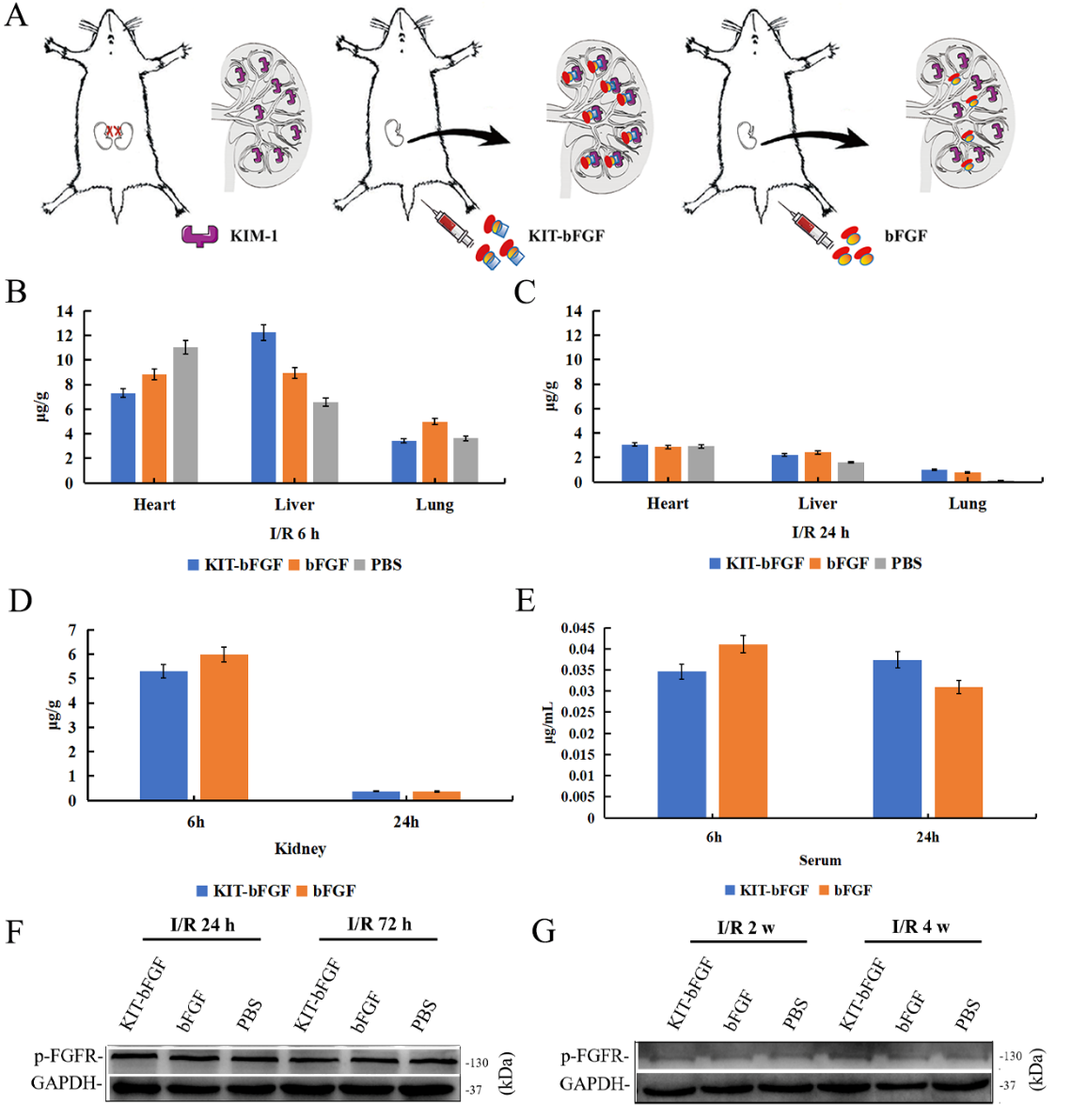
**

**Supplementary Figure 1.** (A) A pattern of fluorescently labeled KIT-bFGF and native bFGF injected into renal I/R rats. (B) Quantitative ELISA assay for bFGF in other tissues at 6 h post-administration; Heart: KIT-bFGF = 7.302 ± 0.558 μg/g, bFGF = 8.812 ± 0.295 μg/g, PBS = 11.021 ± 2.935 μg/g; Liver: KIT-bFGF = 12.223 ± 0.213 μg/g, bFGF = 8.936 ± 0.998 μg/g, PBS = 6.592 ± 0.051 μg/g; Lung: KIT-bFGF = 3.415 ± 0.094 μg/g, bFGF = 4.979 ± 0.744 μg/g, PBS = 3.626 ± 1.001 μg/g. Data are presented as mean ± SD, N = 6. (C) Quantitative ELISA assay for bFGF in other tissues at 24 h post-administration; Heart: KIT-bFGF = 3.060 ± 0.268 μg/g, bFGF = 2.850 ± 0.281 μg/g, PBS = 2.909 ± 0.151 μg/g; Liver: KIT-bFGF = 2.205 ± 0.093 μg/g, bFGF = 2.415 ± 0.282 μg/g, PBS = 1.609 ± 0.128 μg/g; Lung: KIT-bFGF = 1.015 ± 0.204 μg/g, bFGF = 0.783 ± 0.065 μg/g, PBS = 0.111 ± 0.099 μg/g. Data are presented as mean ± SD, N = 6. (D) Quantitative ELISA assay for bFGF in normal rats’ kidney. 6 h: KIT-bFGF = 5.294 ± 0.220 μg/g, bFGF =5.979 ± 2.261 μg/g, 24 h: KIT-bFGF = 0.374 ± 0.013 μg/g, bFGF = 0.366 ± 0.092 μg/g. Data are presented as mean ± SD, N = 3. (E) Quantitative ELISA assay for bFGF in normal rats’ serum. 6 h: KIT-bFGF = 0.035 ± 0.004 μg/mL, bFGF = 0.041 ± 0.004 μg/mL, 24 h: KIT-bFGF = 0.037 ± 0.001 μg/mL, bFGF = 0.031 ± 0.001 μg/mL. Data are presented as mean ± SD, N = 3. (F-G) Western blot assay was used to detect the expression of phospho-FGFR in ischemic kidneys.


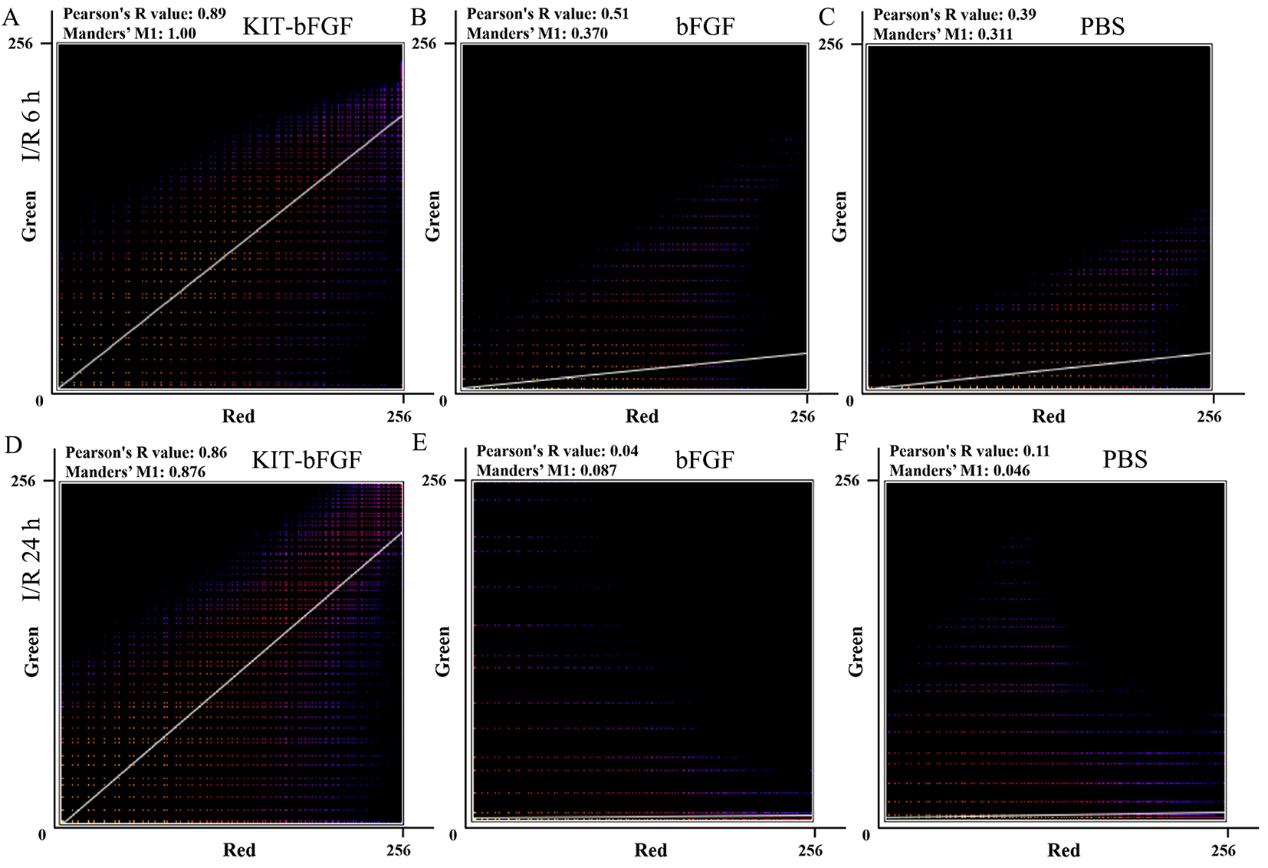


**Supplementary Figure 2.** Statistical of colocalization between FGF2 (bFGF) and KIM-1. The x axis means intensity of red fluorescence (KIM-1), the y axis means intensity of green fluorescence (FGF2), the oblique lines indicate colocalization levels. The closer the slope of the line is to 1, the more colocalization of KIM-1 and FGF2. Pearson's R value means Pearson’s correlation coefficient (PCC), its value is between 1 and -1, 1 means perfect correlation, -1 indicates complete exclusion, and zero indicates random relationship. Manders’ M1 means Manders' Colocalization Coefficients (MCC), which represents the proportion of colocalization of FGF2 and KIM-1 protein to total KIM-1 protein. The closer the value is to 1, the more colocalization area. (A-C) At 6 h post-injection, PCC: KIT-bFGF = 0.89, bFGF = 0.51, PBS = 0.39, MCC: KIT-bFGF = 1.00, bFGF = 0.370, PBS = 0.311; (D-F) At 24 h post-injection, PCC: KIT-bFGF = 0.86, bFGF = 0,04, PBS = 0.11, MCC: KIT-bFGF = 0.876, bFGF =0.087, PBS = 0.046.
